# Renal tubular acidosis with hyperchloremic acidosis: harmless with a sting?

**DOI:** 10.1186/s13054-015-1038-y

**Published:** 2015-08-28

**Authors:** Patrick M. Honore, Rita Jacobs, Inne Hendrickx, Elisabeth De Waele, Viola Van Gorp, Herbert D. Spapen

**Affiliations:** ICU Department, UZ Brussel—VUB University, 101 Laarbeeklaan, 1090 Jette, Brussels, Belgium

Brunner et al. [1] showed a higher than previously described prevalence of renal tubular acidosis (RTA) in critically ill patients with hyperchloremic metabolic acidosis (HMA). They elegantly demonstrated that this condition often remains unrecognized owing to the simultaneous presence of metabolic alkalosis, mainly attributed to low plasma albumin levels, and was not associated with increased morbidity or mortality. HMA was thought to result from altered renal chloride handling as seen in RTA and was considered a nonharmful physiological response.

In contrast with the study of Brunner et al., HMA is most often exogenously induced by too liberal infusion of chloride-containing intravenous fluids for hydration and resuscitation purposes [2]. HMA indeed has no proven impact on ICU and hospital mortality but may precipitate acute kidney injury. It could be assumed that a combination of HMA and RTA only guarantees a relatively stable but fragile acid–base equilibrium with preserved renal function provided the external chloride load remains limited. Excess or overzealous administration of chloride may well precipitate or exacerbate acute kidney injury in these patients. Conversely, early identification of the HMA/RTA combination strongly pleads against a liberal chloride infusion policy and may eventually offer kidney protection!

## Abbreviations

HMA: Hyperchloremic metabolic acidosis
RTA: Renal tubular acidosis
